# Clinical and Immunopathological Features of Moyamoya Disease

**DOI:** 10.1371/journal.pone.0036386

**Published:** 2012-04-27

**Authors:** Runhua Lin, Zeyu Xie, Jianfa Zhang, Hongwu Xu, Hang Su, Xuerui Tan, Dongping Tian, Min Su

**Affiliations:** 1 Institute of Clinical Pathology & Department of Pathology, Shantou University Medical College, Shantou, Guangdong, People's Republic of China; 2 Second Affiliated Hospital of Shantou University Medical College, Shantou, Guangdong, People's Republic of China; 3 First Affiliated Hospital of Shantou University Medical College, Shantou, Guangdong, People's Republic of China; 4 The Judicial Critical Center, Shantou University Medical College, Shantou, Guangdong, People's Republic of China; St Michael's Hospital, University of Toronto, Canada

## Abstract

**Background:**

Moyamoya disease (MMD) is a cerebrovascular disease characterized by progressive stenosis or occlusion of the terminal portion of internal carotid arteries and the formation of a vascular network at the base of the brain. The pathogenesis of MMD is still unclear.

**Methodology/Principal Findings:**

We retrospectively analyzed clinical data for 65 consecutive patients with MMD in our institutions and evaluated the histopathological and immunohistochemical findings of intracranial vessels from 3 patients. The onset age distribution was found to have 1 peak at 40–49 year-old age group, no significant difference was observed in the female-to-male ratio (F/M = 1.2). Intracranial hemorrhage was the predominant disease type (75%). Positive family history was observed in 4.6% of patients. Histopathological findings were a narrowed lumen due to intimal fibrous thickening without significant inflammatory cell infiltration, and the internal elastic lamina was markedly tortuous and stratified. All 3 autopsy cases showed vacuolar degeneration in the cerebrovascular smooth muscle cells. Immunohistochemical study showed the migration of smooth muscle cells in the thickened intima, and aberrant expression of IgG and S100A4 protein in vascular smooth muscle cells. The Complement C3 immunoreactivity was negative.

**Conclusion/Significance:**

This study indicated that aberrant expression of IgG and S100A4 protein in intracranial vascular wall of MMD patients, which suggested that immune-related factors may be involved in the functional and morphological changes of smooth muscle cells, and finally caused the thickened intima. A possible mechanism is that deposits of IgG in the damaged internal elastic lamina may underlie the disruption of internal elastic lamina, which facilitated S100A4 positive SMCs migrated into intima through broken portions of the internal elastic lamina, resulting in lumen stenosis or occlusion, leading to compensatory small vessels proliferation.

## Introduction

Moyamoya disease (MMD), also known as spontaneous occlusion of the circle of Willis, is a cerebrovascular disease characterized by progressive stenosis or occlusion of the terminal portion of internal carotid arteries [1]. The abnormal vascular networks (Moyamoya vessels) at the base of the brain act as collateral channels because of ischemic changes occurring in the brain tissue. These fragile vascular networks resemble a “puff of smoke” (Japanese: Moyamoya) on angiography imaging and hence the disease name [2].

This clinical disease was first reported in Japan as hypogenesis of the bilateral internal carotid arteries [3]. MMD occurs predominantly but not exclusively in Japan. The incidence is high in East Asian countries [4] such as Japan and Korea. A 2003 survey found the total number of patients treated in Japan estimated at 7700 [5], an almost 100% increase during the decade (3,900 in 1994). The female-to-male ratio was 1.8, and 12.1% of patients had a family history of the disease. The crude prevalence was calculated as 6.03 per 100,000 people, and in 2003, the annual rate of newly diagnosed cases was 0.54 per 100,000 people. However, the epidemiological features of MMD in mainland China have not been reported officially.

Patients with MMD show 3 disease types: cerebral ischemia (transient ischemic attack and infarction), hemorrhage (intracerebral, intraventricular and subarachnoid), and no symptoms. MMD occurs in both children and adults, but the clinical features differ. Most pediatric patients experience transient ischemic attack (TIA) or cerebral infarction, whereas, about half of adult patients experience intracranial hemorrhage [6].

The pathogenesis of MMD is still unclear, although extensive studies have been carried out all over the world. Pathology examinations of intracranial arteries at autopsy have shown the outer diameters of the affected internal carotid artery terminations markedly diminished. Microscopically, the arteries of the circle of Willis show narrowed lumen, fibrocellular intimal thickening, marked tortuousness of internal elastic lamina and attenuation of media, with thickened intima composed of smooth muscle cells.

With the improvement of diagnostic techniques, the number of reported cases of MMD have increased, once symptomatic, insufficient cerebral blood flow or rupture of the fragile collaterals may cause stroke or hemorrhage, thus resulting in severe neurological dysfunction or death.

Here, we present a retrospective analysis of cases of MMD in our institutions, pathology and immunohistochemical results of 3 autopsy cases, to describe the demographic profile, clinical features, and histopathological changes of intracranial vessels in patients with MMD.

## Materials and Methods

### 1. Cases

We collected records for 38 patients with MMD treated at the First Affiliated Hospital, and 24 treated at the Second Affiliated Hospital, Shantou University Medical College, from April 2005 to September 2011. In addition, we obtained material for 3 autopsy cases of MMD from the Judicial Critical Center, Shantou University Medical College. All patients had undergone CT scan of the head and cerebral angiography to confirm the diagnosis. The diagnosis of MMD was based on the guidelines established by the Research Committee on Moyamoya Disease (Spontaneous Occlusion of the Circle of Willis) of the Ministry of Health and Welfare of Japan [7]. All participants involved in our study were given written informed consents. Our research has been approved by Ethical Committee of Shantou University Medical College. To compare the clinical features of MMD in different districts, we also obtain articles about MMD written in English (published between 1997 and 2012) through Medline via PubMed (4089 cases, including 65 cases in our institutions).

### 2. Autopsy specimens

At autopsy, the specimens of brain vessels were obtained from 3 adult patients with MMD and fixed in 10% formalin solution. Sections with the circle of Willis and its major branches were dehydrated and embedded in paraffin then sections 4 µm thick were cut from paraffin blocks and deparaffinized in xylene, rehydrated in a descending series of ethanol concentrations, and prepared for histopathological and immunohistochemical studies.

### 3. Antibodies

Rabbit Anti-Human S100A4 polyclonal antibody (S100A4; 1∶100; Zhongshan Golden Bridge), Mouse Anti-Human Actin monoclonal antibody (α-SMA; Maixin Biotechnology), and Mouse Anti-Human IgG monoclonal antibody (IgG; 1∶3000; Santa Cruz) were used as the primary antibodies in this study. As secondary antibody, poly peroxidase-anti-mouse/rabbit IgG (Shanghai Long Island) was used.

### 4. Immunohistochemistry

After being blocked with 3% H_2_O_2_, each section was incubated overnight at 4°C with the desired primary antibodies, rinsed 3 times in phosphate buffered saline (PBS; pH 7.2) and incubated with secondary antibody at 37°C for 30 minutes. After 3 more rinses in phosphate buffered saline, the sections underwent color development for 1 minute at room temperature in a substrate medium containing 3,3-diaminobenzidine. After washed with distilled water, the sections were counterstained with hematoxylin.

### 5. Statistical analysis

Statistical analysis involved use of SPSS 13.0 (SPSS Inc., Chicago, IL). A two-tailed *P*<0.05 was considered statistically significant.

## Results

### 1. Demographic data

We identified 65 patients with MMD in our institutions in Chaoshan District. There were 29 men and 36 women. No significant difference was observed in the gender distribution. The ratio of female to male patients was 1.2. The mean age at symptom onset was 41.7±11.4 (range, 7–73) years (Table 1). The mean onset age in male patients (42.4±10.9) almost equaled to female patients (41.2±12.0). The primary symptoms included headache (29/65), vomiting (28/65), and dizziness (21/65). 63 (97%) patients were adults over the age of 18, and only 2 (3%) pediatric patients. Onset age distribution of the 65 patients was found to have 1 peak at 40–49 year-old age group, and patients in their thirties and forties accounted for the majority (72%). Female patients followed the same pattern in age distribution, however, male patients were frequently observed in their thirties. Positive family history was observed in 4.6% of patients (3/65).

**Table 1:** Summary of patients with MMD in Chaoshan District.^a^

	No. of cases(%)	Mean age	Primary symptoms
Male	29(44.6)	42.4±10.9	Headache 14/29; vomiting 10/29;
			weakness in extremities 9/29;
			dizziness 8/29; etc.
Female	36(55.4)	41.2±12.0	Vomiting 18/36; headache 15/36;
			dizziness 13/36; etc.
Total	65	41.7±11.4	Headache 29/65; vomiting 28/65;
			dizziness 21/65; etc.

a No: number.

With comparison, high female-to-male ratios (about 2∶1) were found in United States and Japan but about a 1∶1 ratio in China (Table 2). The age distribution was bimodal in all areas, including Asian countries. Positive family history was frequently found in patients from Asian countries.

**Table 2:** Comparison of MMD in different districts.^a^

Districts	No. of cases	F/M	Age distribution	Family history
China [10], [17]	1099*	1.02	2 peaks	4.3%
Japan [5], [9]	2416	1.84	2 peaks	10.8%
South Korea [15]	334	1.46	2 peaks	1.5%
United States [8], [13]–[14]	154	2.35	2 peaks	NA
Total	4089	1.53	2 peaks	NA

a No: number; F: female; M: male; NA: not available;

* including 65 MMD patients in our institutions.

### 2. Disease type

**Figure 1:**
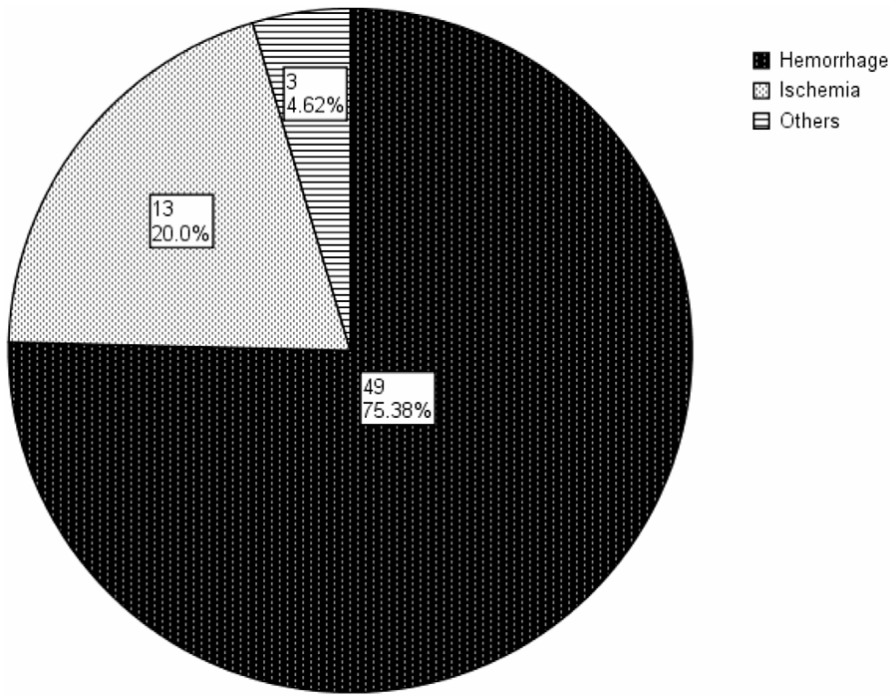
Disease pattern of MMD in Chaoshan District.

**Figure 2:**
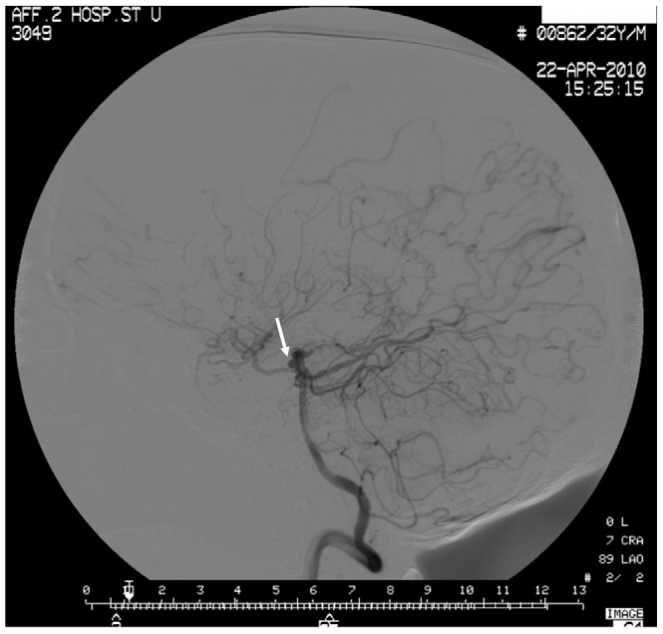
Digital subtraction angiogram (DSA)showing stenosis of the distal portion of the ICA (white arrow).

It is obvious that hemorrhage was much more common in MMD patients (Figure 1). Intracranial hemorrhage affected 75% (49/65) of patients with MMD. Cerebral ischemia occurred in 20% (13/65) of the patients. In the 49 hemorrhagic cases, intracerebral hemorrhage (ICH) occurred in 30 cases (61%), intraventricular hemorrhage (IVH) in 14 cases (29%), and subarachnoid hemorrhage (SAH) in 5 cases (10%). Internal carotid arteries and middle cerebral arteries were frequently affected. Digital subtraction angiogram of a 32-year-old man showing marked stenosis of the distal portion of the ICA (Figure 2).

### 3. Histopathology results for 3 cases

**Figure 3:**
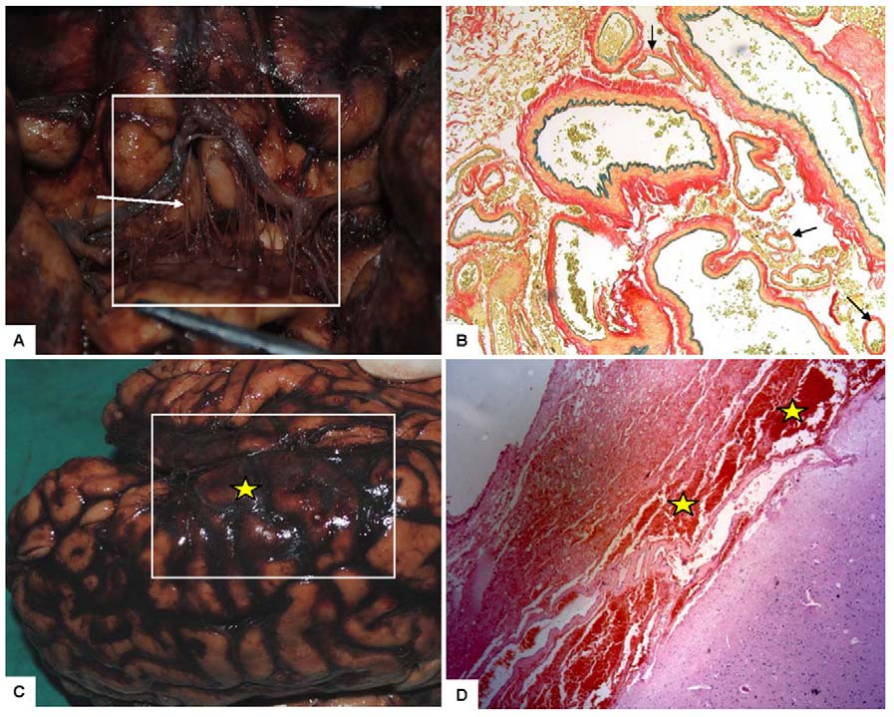
Macroscopical and microscopical findings at autopsy. A: malformation of the vascular network around the proximal middle cerebral artery (white arrow); B: fine vascular network indicated by black arrows (Ponceau/Victoria blue staining, ×100); C: subarachnoid hemorrhage in the right parieto-temporal area; D: subarachnoid hemorrhage in right parietal lobe (H&E staining, ×100); yellow stars indicate the hemorrhagic area.

All 3 autopsy cases of MMD showed diffuse subarachnoid hemorrhage on the brain surface. Fine vascular networks were observed in the proximal of the bilateral middle cerebral arteries and around the pons (Figure 3). Hemorrhage was found in vascular networks but not aneurysms. The tonsils of the cerebellum had no obvious impression.

**Figure 4:**
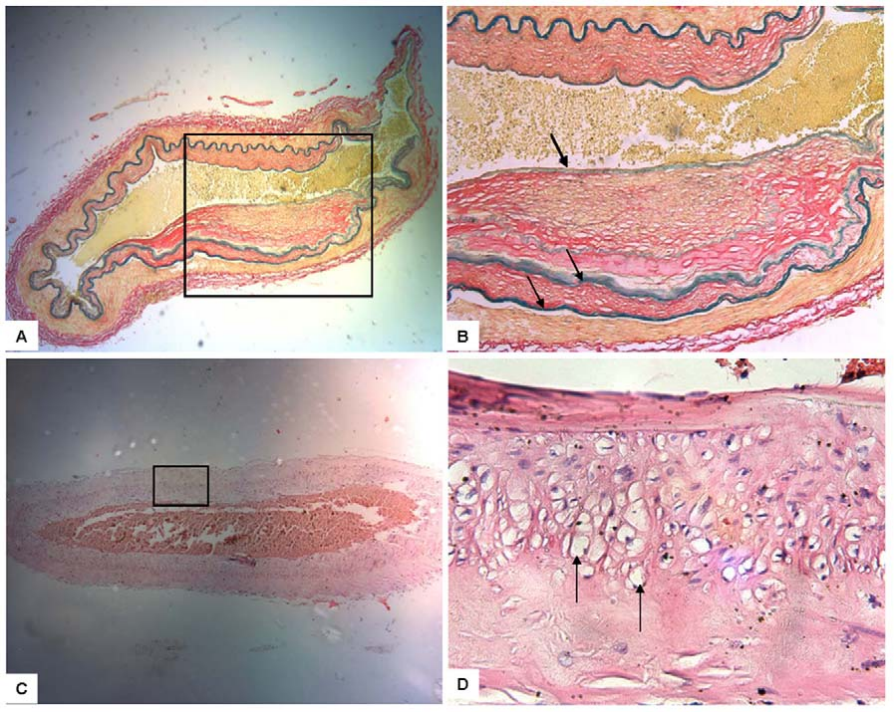
Photomicrographs of brain vessels in MMD patients. A and B: narrow lumen with intimal thickening and attenuation of media in middle cerebral artery (Ponceau/Victoria blue staining, A: ×40, B: ×100), black arrows indicate stratified internal elastic lamina, Figure 4B is an enlarged region (box) of Figure 4A; C and D: vacuolar degeneration (black arrows) in cerebrovascular smooth muscle cells (H&E staining, C: ×40, D: ×100), Figure 4D is an enlarged region (box) of Figure 4C.

Histopathology findings of intracranial vessels revealed a narrowed lumen due to intimal fibrous thickening without significant inflammatory cell infiltration, the vascular wall was thickened. The internal elastic lamina was markedly tortuous and stratified (Figure 4A & 4B). The internal elastic lamina of affected arteries was tortuous, ruptured, straight or multilayered. Interestingly, all 3 autopsy cases showed vacuolar degeneration in the cerebrovascular smooth muscle cells (Figure 4C & 4D).

### 4. Immunohistochemistry results

**Figure 5:**
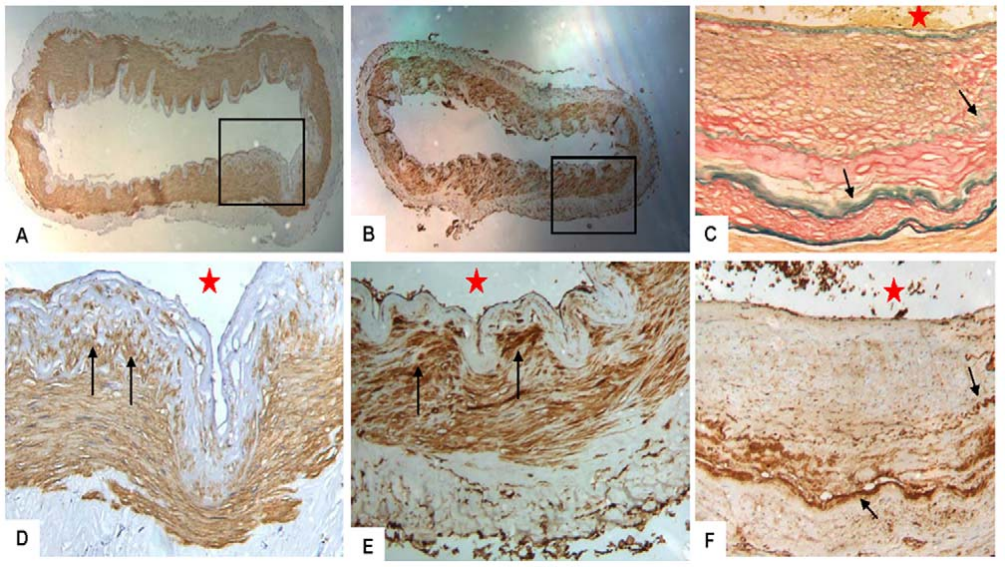
Immunohistochemical study of intracranial arteries. A and D: immunoreactivity indicating that α-SMA is detected in the thickened intima of MCA of patients with MMD (black arrows), Figure 5D is an enlarged region (box) of Figure 5A; B and E: immunoreactivity indicating that S100A4 protein is detected in the thickened intima and media smooth muscle cells (black arrows), Figure 5E is an enlarged region (box) of Figure 5B; F: vascular smooth muscle cells, especially the damaged internal elastic lamina, showed intense immunoreactivity of IgG (black arrows); C: the same area of Figure 5F, black arrows indicate the stratified internal elastic lamina (Ponceau/Victoria blue staining). The red stars indicate lumen of the blood vessels. (A, B) ×40, (C, D, E, F) ×200.

The thickened intima of middle cerebral arteries were positive for α-SMA (Figure 5A & 5D). S100A4 protein immunoreactivity was strongly positive in the thickened intima and media (Figure 5B & 5E). The expression of IgG was positive in intracranial vascular smooth muscle cells, especially in the damaged internal elastic lamina (Figure 5F). The Complement C3 immunoreactivity was negative.

### 5. Follow-up

**Figure 6:**
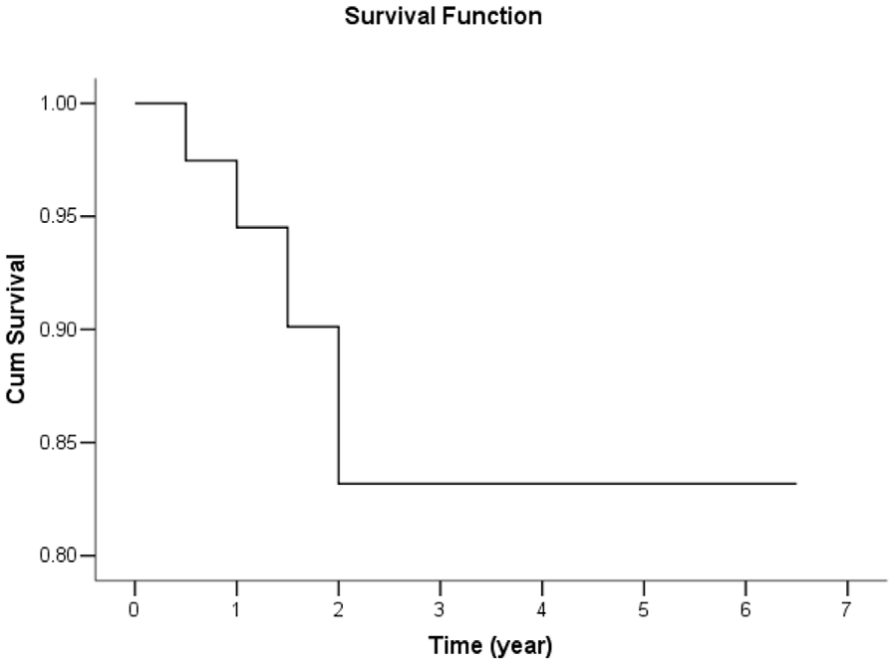
Kaplan-Meier plot for stroke-free survival after treatment.

For the 62 cases of MMD from our institutions, the median follow-up after the first diagnosis (n = 40) was 24.8 months (range 6–78 months); 22 patients could not be contacted for data collection. During the follow-up, 4 strokes occurred in 4 patients (3 hemorrhagic and 1 ischemic). All 4 strokes occurred during the first 2 years after conservative treatment. The 2-year stroke-free survival rate was 83%, with no changes in the 5-year survival rate (Figure 6).

## Discussion

This study showed no significant difference in the female-to-male ratio (F/M = 1.2) in MMD patients. Previous reports [5], [8] showed the female dominance in patients with MMD, in the United States, the ratio of female to male was 2.5, and 1.8 in Japan.

In the Japan series [5], [9] there are 2 peaks in onset age distribution. In our cases, however, there was only 1 peak in the 40–49 year-old age group. The reason for the lack of a peak in the pediatric group in our institutions was not clear. An important factor contributed to this phenomenon is that some pediatric MMD patients were not diagnosed in time. Generally, pediatric patients with MMD experienced ischemic symptoms, however, because of their young age (less than 10 years old), they usually could not expressed their symptoms clearly. In addition, the parents' educational background and socioeconomic status may also have influence on their decisions to take the diagnostic tests or not.

As mentioned above, intracranial hemorrhage as clinical manifestations were much more common than ischemia in our 65 cases. Because 97% patients were adults, our result supported that most adult MMD patients experienced intracranial hemorrhage. The 2-year stroke-free survival rate was 83%, with no changes in the 5-year survival rate, so the critical period is 2-year-period after the first onset.

Since MMD was initially described in Japan, it has been reported frequently, especially in East Asia. In China, MMD has been described frequently in the literature. Recently, a study of the clinical features of MMD in China was published [10]; however, the number of cases in a single institution are too few for in-depth study; many cases that occur in mainland China may be neglected or reported by case reports. To make comparisons among different districts, we searched the literature [5], [8]–[17] to comprehensively compare cases of MMD worldwide.

**Figure 7:**
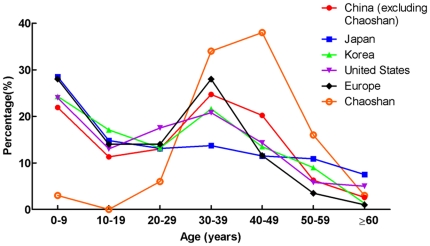
Onset age distribution of MMD in different districts.

Previous study [18] showed a female dominance in patients with MMD. We found about a 2∶1 ratio for the United States and Japan but about a 1∶1 ratio in China (Table 2). The positive family history was frequently found in patients from Asian countries. The age distribution was bimodal in all areas, including Asian countries. The onset age was predominantly 0–9 years and 30–39 years (Figure 7).

Histopathological changes of the distal portion of the internal carotid arteries and the beginning of the middle cerebral arteries are fibrocellular thickening of the intima and markedly wavy, often ruptured or duplicated elastic lamina. These findings of intracranial vessels are consistent with previous reports [7], [19]–[23]. The changes in intracranial vessels may increase the fragility of vessels, which are susceptible to bleeding when blood pressure fluctuates.

Immunohistochemical examination showed α-SMA-positive cells in the thickened intima, which suggested that vascular smooth muscle cells migrated into the intima and proliferated. Strikingly, S100A4 protein and IgG immunoreactivity were strongly positive in the media and intima of the major arteries in MMD patients.

The deposits of IgG in vascular wall may underlie the disruption of internal elastic lamina, facilitating S100A4-positive SMCs migrate into intima. S100A4 protein belongs to the S100 family of calcium binding proteins. The role of S100A4 in tumor progression and metastasis is well documented [24], [25]. Recently, implications of S100A4 protein in various non-malignant pathological conditions have been demonstrated [26]. Tight association between S100A4 expression and smooth muscle cells phenotypic modulation in porcine and human coronary arteries was also demonstrated. The aberrant expression of S100A4 protein is associated with upregulation SMCs proliferative and migratory activities [27]. So, we speculate that MMD might be caused by immune-related factors and then internal elastic lamina disrupted, smooth muscle cells in the media changed morphologically and functionally, by, for example, vacuolar degeneration in the smooth muscle cells and migrated into intima, resulting in lumen stenosis or occlusion.

In conclusion, we performed a retrospective analysis of patients with MMD, and described the clinical features and outcomes. Histopathological and immunohistochemical findings of intracranial vessels from 3 autopsy cases suggested that immune-related factors may involved in the functional and morphological changes of smooth muscle cells, which finally caused the thickened intima.
